# MIA: non-targeted mass isotopolome analysis

**DOI:** 10.1093/bioinformatics/btw317

**Published:** 2016-06-06

**Authors:** Daniel Weindl, Andre Wegner, Karsten Hiller

**Affiliations:** Luxembourg Centre for Systems Biomedicine, University of Luxembourg, L-4362 Esch-sur-Alzette, Luxembourg

## Abstract

**Summary:** MIA detects and visualizes isotopic enrichment in gas chromatography electron ionization mass spectrometry (GC–EI-MS) datasets in a non-targeted manner. It provides an easy-to-use graphical user interface that allows for visual mass isotopomer distribution analysis across multiple datasets. MIA helps to reveal changes in metabolic fluxes, visualizes metabolic proximity of isotopically enriched compounds and shows the fate of the applied stable isotope labeled tracer.

**Availability and Implementation:** Linux and Windows binaries, documentation, and sample data are freely available for download at http://massisotopolomeanalyzer.lu. MIA is a stand-alone application implemented in C ++  and based on Qt5, NTFD and the MetaboliteDetector framework.

**Contact:** karsten.hiller@uni.lu

## 1 Introduction

Stable isotope assisted metabolomics is widely applied to analyze metabolism and to determine reaction mechanisms (Buescher *et al.*, 2015). However, current approaches are usually highly targeted and only take into account a small set of metabolites. We recently demonstrated the potential of non-targeted stable isotope labeling in the analysis of hypoxic cancer cells to detect hypoxia-induced metabolic flux changes in a non-targeted manner (Weindl *et al.*, 2016).

There have been tools available for the non-targeted and quantitative detection of isotopic enrichment in complex samples analyzed by GC-MS (Hiller *et al.*, 2013) or LC-MS (Bueschl *et al.*, 2012; Capellades *et al.*, 2016; Chokkathukalam *et al.*, 2013; Creek *et al.*, 2012; Huang *et al.*, 2014). These tools provide the isotopic enrichment of all detected compounds in the form of mass isotopomer distributions (MIDs), which represent the relative abundances of the different isotopic isomers (isotopologues) grouped by their masses. However, such metabolome-wide stable isotope labeling data has barely been analyzed to extract biological information in a truly non-targeted manner. One reason is, that there is still a lack of adequate software to facilitate the analysis of such datasets. Without proper data analysis tools, a global mass isotopolome analysis and its biological interpretation is tedious.

**Fig. 1.**
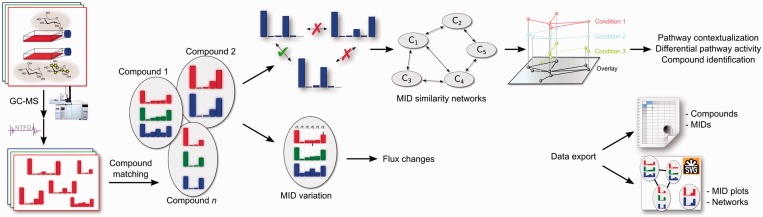
MIA workflow. After performing a stable isotope labeling experiment, isotopic enrichment is detected and quantified in a non-targeted manner. Labeled compounds are matched across all datasets and MIDs are visualized. Data can be filtered and analyzed by changes in MIDs which indicate metabolic flux changes. MID-similarity can be visualized to reveal metabolic proximity of the isotopically enriched compounds to aid compound identification or to reveal their biosynthetic pathways. Finally, data can be exported either as spreadsheet or vector graphics for further use (Color version of this figure is available at *Bioinformatics* online.)

Until recently, there was neither free nor commercial software available that sufficiently supported the biological analysis of global stable isotope labeling datasets beyond the mere detection of isotopic enrichment. To this end, we developed MIA, an easy-to-use software tool to determine, visualize and analyze mass isotopomer distributions across multiple GC–EI-MS datasets in a non-targeted manner (Fig. 1).

## 2 Features

MIA, the *Mass Isotopolome Analyzer*, provides the following features:

- Non-targeted determination of mass isotopomer distributions
- Matching detected compounds across different datasets
- Visualization of MIDs of all mass-spectrometric fragments
- Analysis of MID changes
- Visualization of compound networks based on MID similarity
- Compound identification using reference libraries
- Export of graphics or spreadsheet data
- Integration with MetaboliteDetector software for further data analysis (http://metabolitedetector.tu-bs.de/)

### 2.1 Data visualization

MIA visualizes the MIDs of the detected fragments as barplots. All detected isotopically enriched mass spectrometric fragments can be analyzed. If replicate measurements are provided, confidence intervals and quality measures of MID determination such as coefficient of determination are provided (Weindl *et al.*, 2015b).

### 2.2 Mass isotopomer abundance variation analysis

MIDs from different datasets can be analyzed for changes in relative mass isotopomer abundance. Such variations are caused by, and are indicative of, altered metabolic fluxes. Therefore, metabolic flux changes are detected in a non-targeted manner (Weindl *et al.*, 2015a, 2016).

### 2.3 MID similarity networks

Usually compounds closely connected within the metabolic network show very similar MIDs (Weindl *et al.*, 2016). MIA exploits this fact for the analysis of stable isotope labeling data: Isotopically enriched compounds can be visualized in form of a network with connectivity based on their pairwise similarity. Due to this network visualization, closely related compounds can easily be detected. Knowledge on metabolic similarity can be valuable for compound identification, addressing a common bottleneck of non-targeted metabolomics.

## 3 Experimental requirements

MIA operates on low resolution GC–EI-MS data in the MetaboliteDetector or the commonly used netCDF format. MIDs are determined from the difference between mass spectra of an isotopically enriched compound and those of the non-enriched compound (Weindl *et al.*, 2015b). Thus, two metabolite extracts need to be measured: One from a stable isotope labeling experiment and one from a label-free experiment.

## 4 Implementation

MIA is implemented in C ++ and Qt5. The MetaboliteDetector (Hiller *et al.*, 2009) library is used for GC–EI-MS data handling, the NTFD library (Hiller *et al.*, 2010, 2013) for non-targeted detection and quantification of stable isotope labeling and the GraphViz library for graph layouts (Gansner and North, 2000). Binaries are available for Linux and Windows operating systems.

## 5 Conclusion

Non-targeted stable isotope labeling analysis is a versatile tool for metabolic research. However, it has not yet reached its full potential due to the lack of appropriate data analysis software. MIA helps the user to quickly visualize and analyze this complex data and to gain new biological insights eventually. A more in-depth description of the implemented workflows and a case study applying the described workflows are available in Weindl *et al.* (2016).
